# Etiology of Bacterial Meningitis Among Children <5 Years Old in Côte d’Ivoire: Findings of Hospital-based Surveillance Before and After Pneumococcal Conjugate Vaccine Introduction

**DOI:** 10.1093/cid/ciz475

**Published:** 2019-09-05

**Authors:** Catherine Boni-Cisse, Sheikh Jarju, Rowan E Bancroft, Nicaise A Lepri, Hamidou Kone, N’zue Kofi, Alice Britoh-Mlan, Flore Sandrine Zaba, Effua Usuf, Peter Sylvanus Ndow, Archibald Worwui, Jason M Mwenda, Joseph N Biey, Bernard Ntsama, Brenda A Kwambana-Adams, Martin Antonio

**Affiliations:** 1 Département de Microbiologie, Université Félix Houphouët Boigny, Abidjan, Côte d’Ivoire;, UFR des Sciences Médicales; 2 Sentinel Site Surveillance Laboratory of Paediatric Bacterial Meningitis and Rotavirus Diarrhoea, Centre Hospitalier Universitair de Yopougon, Abidjan, Côte d’Ivoire; 3 World Health Organization (WHO) Collaborating Centre for New Vaccines Surveillance, Medical Research Council Unit The Gambia at London School of Hygiene and Tropical Medicine, Fajara, Banjul, The Gambia; 4 WHO Country Office, Abidjan, Côte d’Ivoire; 5 WHO Regional Office for Africa, Brazzaville, Republic of Congo; 6 WHO Intercountry Support Team, Ouagadougou, Burkina Faso; 7 Microbiology and Infection Unit, Warwick Medical School, University of Warwick, Coventry, United Kingdom

**Keywords:** Côte d’Ivoire, pediatric, meningitis, pneumococcus, *Haemophilus influenzae*

## Abstract

**Background:**

Bacterial meningitis remains a major disease affecting children in Côte d’Ivoire. Thus, with support from the World Health Organization (WHO), Côte d’Ivoire has implemented pediatric bacterial meningitis (PBM) surveillance at 2 sentinel hospitals in Abidjan, targeting the main causes of PBM: *Streptococcus pneumoniae* (pneumococcus), *Haemophilus influenzae*, and *Neisseria meningitidis* (meningococcus). Herein we describe the epidemiological characteristics of PBM observed in Côte d’Ivoire during 2010–2016.

**Methods:**

Cerebrospinal fluid (CSF) was collected from children aged <5 years admitted to the Abobo General Hospital or University Hospital Center Yopougon with suspected meningitis. Microbiology and polymerase chain reaction (PCR) techniques were used to detect the presence of pathogens in CSF. Where possible, serotyping/grouping was performed to determine the specific causative agents.

**Results:**

Overall, 2762 cases of suspected meningitis were reported, with CSF from 39.2% (1083/2762) of patients analyzed at the WHO regional reference laboratory in The Gambia. In total, 82 (3.0% [82/2762]) CSF samples were positive for bacterial meningitis. Pneumococcus was the main pathogen responsible for PBM, accounting for 69.5% (52/82) of positive cases. Pneumococcal conjugate vaccine serotypes 5, 18C, 19F, and 6A/B were identified post–vaccine introduction. Emergence of *H. influenzae* nontypeable meningitis was observed after *H. influenzae* type b vaccine introduction.

**Conclusions:**

Despite widespread use and high coverage of conjugate vaccines, pneumococcal vaccine serotypes and *H. influenzae* type b remain associated with bacterial meningitis among children aged <5 years in Côte d’Ivoire. This reinforces the need for enhanced surveillance for vaccine-preventable diseases to determine the prevalence of bacterial meningitis and vaccine impact across the country.

## Teaser

Longitudinal sentinel surveillance at 2 hospitals within Abidjan, Côte d’Ivoire, during 2010–2016 revealed that *Streptococcus pneumoniae* was the predominant pathogen responsible for pediatric bacterial meningitis before and after the introduction of the 13-valent pneumococcal conjugate vaccine.

The 26 countries that are within the extended meningitis belt across sub-Saharan Africa, including Côte d’Ivoire, experience the heaviest burden of bacterial meningitis worldwide. In 2012, a meningitis outbreak affecting 10 countries with >11 000 cases and 960 deaths occurred [1]. *Neisseria meningitidis* (meningococcus) was the pathogen responsible for this outbreak, and Côte d’Ivoire was 1 country that was affected, with a reported 399 suspected cases and 49 deaths due to meningococcus, resulting in a case fatality rate of 12.3% [1, 2]. Bacterial meningitis epidemics have been reported in Africa for more than a century, with the highest rates of mortality observed in children <5 years old [2]. Historically, up to 80% of bacterial meningitis cases are caused by *Streptococcus pneumoniae* (pneumococcus), *Haemophilus influenzae*, and meningococcus [3, 4].

Consequently, in 2008, the World Health Organization (WHO) started standardizing invasive bacterial vaccine-preventable disease (IB-VPD) surveillance, with the aim of ensuring that all sentinel hospitals have access to advanced molecular laboratory diagnostic tests for meningitis [5]. As part of this, the WHO established regional reference laboratories (RRLs). One such RRL is based at the Medical Research Council Unit The Gambia at the London School of Hygiene and Tropical Medicine (MRCG at LSHTM), which supports pediatric bacterial meningitis (PBM) surveillance from 17 sentinel sites across West Africa [5]. This includes Côte d’Ivoire, which has a population of approximately 26 million, a human immunodeficiency virus prevalence of 2.8% in adults aged 15–49 years, and an estimated 10 million children <14 years of age who are most at risk of bacterial meningitis infections [6, 7].

In 2009, Côte d’Ivoire introduced the *H. influenzae* type b (Hib) vaccine for children aged 8, 12, and 16 weeks. However, the civil war from 2010 to 2011 led to a reduction in routine immunization coverage rates from approximately 85% to 62% [8, 9]. Since then, the Hib-containing vaccine coverage rate has rapidly improved, with WHO/United Nations Children’s Fund estimates at 84% for 2017 [10]. In 2013, the 13-valent pneumococcal conjugate vaccine (PCV13), which targets 13 invasive pneumococcal serotypes, was introduced, and coverage was estimated at 99% in 2017 [10, 11]. Additionally, in 2014 Côte d’Ivoire conducted vaccination campaigns using the meningococcal conjugate vaccine (MenAfriVac), and it is expected that the country will soon apply for funding from Gavi, the Vaccine Alliance, to introduce MenAfriVac into their routine Expanded Programme on Immunization [12, 13].

With support from the WHO and the MRCG at LSHTM, Côte d’Ivoire has implemented surveillance at 2 sentinel hospitals in its capital city, Abidjan, with the aim to provide comprehensive surveillance data on children <5 years old with suspected meningitis, over a 7-year period. Here, we present a summary of this surveillance data for the period 2010–2016.

## MATERIALS AND METHODS

### Surveillance Design and Target Population

Surveillance of children <5 years old with suspected meningitis, who were admitted to either Abobo General Hospital or University Hospital Center Yopougon based in the Abidjan suburbs, was carried out from 2010 through 2016. Children were referred by pediatricians at primary and secondary healthcare facilities to these sentinel hospitals based on their place of residence. Additionally, some children were admitted directly by the hospitals after presenting at the emergency room. Suspected meningitis cases were identified using the WHO standard case definition by the rapid development of a fever with axillary and rectal temperatures >38°C or >38.5°C, respectively, combined with at least 1 of the following clinical symptoms: reduced level of consciousness, meningismus (stiff neck), photophobia, bulging fontanelle (infants), and convulsions or partial seizures [14].

### Cerebrospinal Fluid Processing at the Sentinel Site

Confirmed bacterial meningitis cases were defined by the identification of bacteria in the cerebrospinal fluid (CSF) [14]. A lumbar puncture was performed and CSF samples were collected from suspected cases following a standard operating procedure developed by the WHO Paediatric Bacterial Meningitis Network [5, 15]. Samples were transported to the sentinel site laboratory for processing within 1 hour of collection. The CSF appearance was recorded, and white blood cell (WBC) counts, protein levels, and glucose levels were measured using methods previously described [15–17].

An aliquot of CSF was centrifuged and the supernatant used in a rapid antigen test with the use of latex agglutination (Pastorex meningitis kit, Bio-Rad) to detect the presence of *S. pneumoniae*, *H. influenzae*, or *N. meningitidis.* Some of the sediment was used for Gram staining, while another loopful was streaked onto Columbia blood agar and chocolate agar plates and incubated overnight. On examination, suspected pneumococcal isolates were confirmed via an optochin test (5 μg optochin disk, Oxoid), and suspected *H. influenzae* and meningococcal isolates underwent an analytical profile index (API NH, bioMérieux). Where possible, serotyping and serogrouping for isolates was performed using various latex agglutination techniques, as previously described [18–20]. Antibiotic susceptibility tests were performed for commonly prescribed antibiotics via disk and Etest diffusion following Clinical and Laboratory Standards Institute guidelines [21]. Shipment of CSF samples to MRCG at LSHTM was carried out in accordance with the International Air Transport Association regulations for infectious substances [22].

### Molecular Analysis of CSF

Once at MRCG at LSHTM, species-specific real-time polymerase chain reaction (PCR) was performed with the following gene targets: *lytA* for *S. pneumoniae*, *hpd* for *H. influenzae*, and *sodC* for *N. meningitidis* [23, 24]. An RNase P assay was used to confirm that samples were of human origin, and cycle threshold (Ct) values ≤36 were considered positive results. A sequential triplex real-time PCR assay to detect 21 pneumococcal capsular serotypes was conducted as previously described [25, 26]. Samples with Ct values >32 were examined by conventional PCR [27]. Targets for *N. meningitidis* included the *sacB*, *synD*, *synE*, *synG*, *xcbB*, and *synF* genes for serogroups A, B, C, W, X, and Y, respectively. For *H. influenzae*, the following genes were screened for serotyping: *acsB* (type a), *bcsB* (Hib), *ccsD* (type c), *dscE* (type d), *ecsH* (type e), and *bexD* (type f), following methods described elsewhere [28]. Ct values of ≤32 were considered positive.

### Statistical Analysis

Surveillance data was recorded by the 2 sentinel hospitals using standardized WHO Regional Office for Africa PBM network case report forms. Here, details of patient demographics, clinical symptoms, and laboratory test results were collected. This information was then logged on a WHO Epi Info–based customized new vaccines surveillance data module. Data cleaning and analysis were performed at the sentinel sites and then sent to national and regional WHO data managers. All data were analyzed using GraphPad Prism 8.1.1 software, and baseline characteristics of the children with suspected meningitis and pathogens detected were summarized using descriptive statistics.

**Ethical Considerations**

Ethical approval was not a requirement in Côte d’Ivoire for routine meningitis surveillance, including drug susceptibility testing of collected isolates, as this is approved within the routine diagnostic algorithm at the Ministry of Health. However, informed consent was sought from caregivers of the surveillance participants. Additionally, the surveillance received overarching ethical approval (SCC1188) by the joint MRCG at LSHTM/The Gambia government ethics board that allowed the analysis of collected West African isolates.

## RESULTS

### Clinical and Demographic Characterization of Enrolled Cases

**Figure 1.**
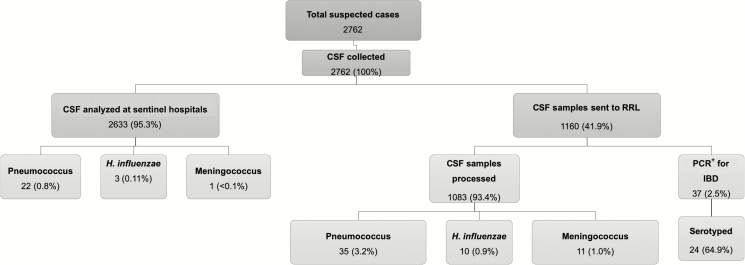
A breakdown of the suspected pediatric bacterial meningitis cases during surveillance in Côte d’Ivoire, 2010–2016. A total of 2762 cerebrospinal fluid (CSF) samples were collected from patients with suspected pediatric bacterial meningitis admitted to 2 sentinel hospitals within Abidjan. Diagnostic tests were performed for most CSF samples at the sentinel hospitals, and 1160 CSF samples were sent to the World Health Organization regional reference laboratory (Medical Research Council Unit The Gambia at the London School of Hygiene and Tropical Medicine) for confirmatory species-specific polymerase chain reaction. Abbreviations: CSF, cerebrospinal fluid; IBD, invasive bacterial disease; PCR, polymerase chain reaction; RRL, regional reference laboratory.

Throughout the surveillance period, 2762 children <5 years old with suspected bacterial meningitis were recruited. Of these, 2743 (99.3%) patients were residents of the Abidjan district, an additional 13 (0.5%) were from the surrounding Lagunes district, and 6 (0.2%) were residents from elsewhere within Côte d’Ivoire. A lumbar puncture was performed for all patients recruited, and CSF samples were collected. In total, 1160 of 2762 (41.9%) CSF samples were sent to the RRL at MRCG at LSHTM for analysis (Figure 1), where 1083 of the 1160 (93.4%) CSF samples were processed to determine if *S. pneumoniae*, *H. influenzae*, or *N. meningitidis* were present (Figure 1).

Details of the clinical and demographic characteristics of the children recruited at either Abobo General Hospital or University Hospital Center Yopougon are summarized in Table 1. More than half of the children recruited were male (1561/2762 [56.5%]), and the median age of patients was 28 (interquartile range, 8–36) months. The greatest number of suspected cases were seen in the eldest children, aged 24–59 months (1271/2762 [46.0%]). Interestingly, 175 of the 2762 (7.1%) patients reported using antibiotics prior to hospital admission and CSF collection. Of the 773 (773/2762 [28%]) patients for whom the outcome was recorded, there was a case fatality rate of 6.2%, accounting for 48 in-hospital deaths.

**Table 1.** Summary of Clinical and Demographic Characteristics of the Study Population

Characteristic	Total		*Streptococcus pneumoniae*		*Haemophilus influenzae*		*Neisseria meningitidis*	
	No.	(%)	No.	(%)	No.	(%)	No.	(%)
Age group, mo								
0–11	890	(32.2)	25	(71.4)	8	(22.9)	2	(5.7)
12–23	597	(21.6)	12	(70.6)	2	(11.8)	3	(17.6)
24–59	1271	(46.0)	20	(66.7)	3	(10.0)	7	(23.3)
Unknown	4	(0.1)	0	(0)	0	(0)	0	(0)
Sex								
Male	1561	(56.5)	37	(63.8)	10	(17.2)	11	(19.0)
Female	1199	(43.4)	20	(83.3)	3	(12.5)	1	(4.2)
Unknown	2	(0.1)	0	(0)	0	(0)	0	(0)
Antibiotic before admission								
Yes	175	(6.3)	2	(33.3)	4	(66.7)	0	(0.0)
No	1134	(41.0)	16	(76.2)	3	(14.3)	2	(9.5)
Unknown	1453	(41.3)	39	(70.9)	6	(10.9)	10	(18.2)
CSF appearance								
Clear	2268	(82.1)	22	(64.7)	6	(17.6)	6	(17.6)
Turbid/cloudy	203	(7.3)	33	(76.7)	6	(14.0)	4	(9.3)
Xanthrochromic	87	(3.1)	1	(100.0)	0	(0.0)	0	(0.0)
Blood-stained	197	(7.1)	1	(25.0)	1	(25.0)	2	(50.0)
Unknown/other	7	(0.3)	0	(0)	0	(0)	0	(0)
WBC count, cells/μL								
≤10	2602	(94.2)	21	(0.8)	8	(0.3)	10	(0.4)
>10 to 100	25	(0.9)	5	(20.0)	1	(4.0)	0	(0.0)
>100	75	(2.7)	31	(42.5)	4	(5.5)	2	(2.7)
Unknown	60	(2.2)	0	(0)	0	(0)	0	(0)
Outcome								
Discharged alive	214	(7.7)	1	(100.0)	0	(0.0)	0	(0.0)
Died	48	(1.7)	2	(100.0)	0	(0.0)	0	(0.0)
Transferred	3	(0.1)	0	(0)	0	(0)	0	(0)
Left against medical advice	28	(1.0)	7	(58.3)	5	(38.5)	0	(0.0)
Pending discharge	480	(17.4)	0	(0.0)	0	(0.0)	1	(8.3)
Unknown	1989	(72.0)	47	(71.2)	7	(10.6)	11	(16.7)
Case type^a^: suspected	2762	(100.0)	57	(2.1)	13	(0.5)	12	(0.4)

Abbreviations: CSF, cerebrospinal fluid; WBC, white blood cell.

^a^Suspected cases include cases that were defined as probable as per World Health Organization case definition guidelines [14].

**Figure 2.**
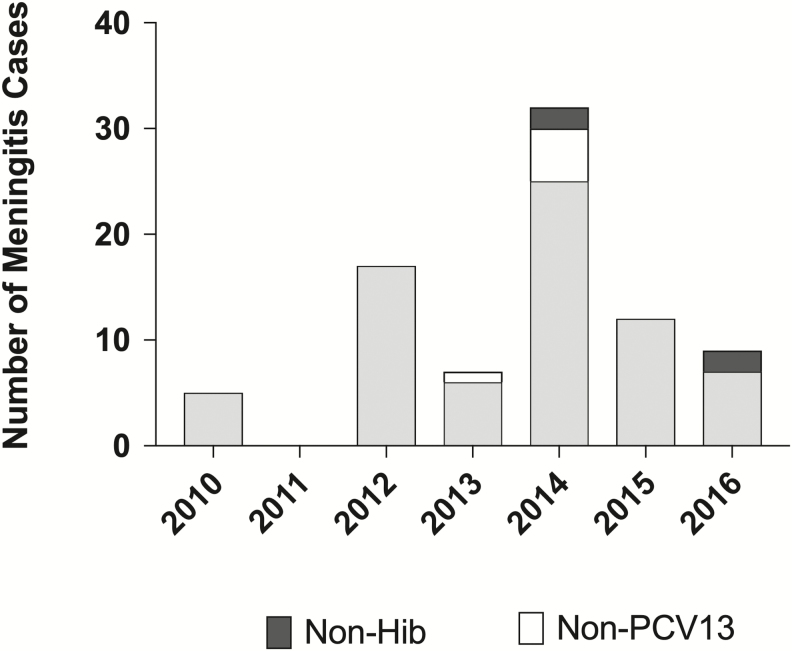
The distribution of confirmed pediatric bacterial meningitis cases within 2 sentinel hospitals in Côte d’Ivoire, 2010–2016. The number of cerebrospinal fluid samples that were positive for *Streptococcus pneumoniae*, *Neisseria meningitidis*, or *Haemophilus influenzae* during each year of surveillance in Côte d’Ivoire are shown. Light gray shading indicates meningitis cases that were caused by bacterial serotype/groups targeted by the meningococcal conjugate vaccines (MenAfriVac, the 13-valent pneumococcal conjugate vaccine [PCV13], and the *H. influenzae* type b [Hib] vaccine). Dark gray shading highlights cases of *H. influenzae* meningitis identified as non–type b, and white shading indicates cases of pneumococcal meningitis caused by serotypes not targeted by PCV13.

The number of suspected bacterial meningitis cases in children aged <5 years varied annually, ranging from 74 cases in 2011 to 726 cases in 2014. The average number of cases annually was 394.6. During the surveillance period, a total of 82 (82/2762 [3.0%]) cases of bacterial meningitis were confirmed from CSF samples collected at the sentinel sites. Of these, 57 (69.5%) were caused by *S. pneumoniae*, 13 (15.8%) by *H. influenzae*, and an additional 12 (14.6%) by *N. meningitidis.* In addition, the highest number of meningitis cases caused by these 3 pathogens was seen in 2014, with 32 cases, accounting for 39% of the total 82 meningitis cases (Figure 2).

### Microbial Identity

The most prevalent pathogen causing PBM during the surveillance period was pneumococcus, accounting for 25 of the 35 (71.4%) meningitis cases seen in children aged 0–11 months, 12 of the 17 (70.6%) cases seen in children 12–23 months, and 20 of the 30 (66.7%) cases seen in children aged 24–59 months. A total of 13 cases of bacterial meningitis were caused by *H. influenzae*, and the majority of these were seen in children from the youngest age group. Additionally, a further 12 cases of bacterial meningitis were caused by *N. meningitidis*, with the majority of these cases occurring in children in the eldest age group.

An interesting observation was that among the 82 patients who had bacterial meningitis, a total of 34 (41.5%) cases provided CSF samples that were clear in appearance. Moreover, of these 34, 22 (64.7%) were identified as positive for pneumococcus. Additionally, a total of 43 of the 82 (52.4%) cases with bacterial meningitis had CSF that appeared turbid or cloudy. Again, of these the majority (33 [76.7%]) were caused by pneumococcal infections. Furthermore, nearly half the total of PBM cases (39/82 [47.5%]) had WBC counts of <10 cells/μL, and 37 cases had WBC counts of >100 cells/μL (Table 1).

### Serotype Distribution

Of the 57 cases of meningitis caused by *S. pneumoniae*, we identified the specific serotype of 16 isolates of the bacterium, collected from 16 children. We found that 9 of these cases were caused by pneumococcal serotypes that are targeted within the PCV13 vaccine including serotypes 5, 18C, 19F, and 6A/6B/6C/6D. On examination with PCR assays, 2 isolates had Ct values >32; thus, the DNA concentration was too low to accurately serotype. A further 2 samples were nontypeable by real-time PCR and were negative for all 21 serotypes targeted. The remaining 3 pneumococcal isolates were serotypes that were non-PCV13, including 18, 12F/12A/12B/44/46, and 33F (Figure 2).

Of the 13 *H. influenzae* cases admitted to the sentinel sites during the surveillance period, we performed serotype analysis on 6 isolates. One-third were positively identified as Hib, another third was determined as *H. influenzae* type a, and the final third were either *H. influenzae* type f or nontypeable by PCR methods (Figure 2). Furthermore, of the 12 cases of *N. meningitidis* observed during 2010–2016, we serogrouped 2 of these, and both were nongroupable by the methods used and were negative for serogroups A, B, C, W, X, and Y.

Interestingly, of the 82 patients with confirmed PBM, 23 (28%) had received at least 1 dose of the Hib vaccine and 12 (14.6%) reported receiving all 3 doses (full course). Two cases of *H. influenzae* were detected among patients who had received vaccines; however, serotype analysis had not been successfully performed for these isolates. The Hib vaccination status of 46 of the 82 (56.0%) patients with PBM was not recorded.

## DISCUSSION

We report findings from the first longitudinal surveillance of PBM cases within Abidjan, Côte d’Ivoire. There have been a number of reports of bacterial meningitis within the meningitis belt previously, but none have focused specifically on Côte d’Ivoire and Abidjan alone [8, 29, 30]. The most common pathogen found to be associated with PBM was *S. pneumoniae*, responsible for 2.1% of the confirmed meningitis cases we analyzed. This finding is in line with previous reports in both The Gambia and China, which found high levels of pneumococcus associated with meningitis [31, 32].

Of the pneumococcal cases that we were able to serotype, we found isolates from 4 PCV13 serotypes (5, 18C, 19F, and 6A/6B/6C/6D) in a total of 9 cases from 2014 through 2016. It is well documented that widespread use of pneumococcal conjugate vaccines (PCVs) leads to a marked reduction in the number of vaccine serotypes carried within the vaccinated and unvaccinated populations [33–35]. Côte d’Ivoire introduced the PCV13 in 2013, with vaccine coverage rates estimated at 99% nationally by 2015 [10]. However, throughout this surveillance period, the PVC13 vaccination status of patients with suspected meningitis was not recorded. In addition, we found 5 cases of non-PCV13 serotypes causing PBM, 4 of which occurred after the introduction of PCV13. A recent United Kingdom–based study found that there has been serotype replacement over 5 winters in pneumococcal carriage of children, whereby vaccine serotypes have been replaced by nonvaccine serotypes [36]. The increased emergence of non-PCV13 serotypes in a post-PCV13 era may be due to changes within the bacterial selection pressure caused by the vaccine, leading to the emergence of greater number of nonvaccine serotypes [37]. Moreover, a recent study by Kwambana-Adams and colleagues found that rapid replacement of PCV serotypes with non-PCV serotypes may diminish the impact of the vaccine [38].

We observed an increased number of cases caused by non-PCV13 serotypes in the post-PCV13 era (after 2013). However, as only 24.5% (14/57) of the pneumococcal meningitis cases were successfully serotyped, it is difficult to conclude that serotype replacement is occurring in Côte d’Ivoire. In addition, it is difficult to make this conclusion using this sentinel surveillance data alone, especially without details of the PCV13 vaccination status of patients. Thus, our data highlight that further work within the country is required to ensure high vaccine coverage that may lead to herd immunity against vaccine serotypes in older children. In addition, further monitoring, reporting, and follow-up of the prevalence of both vaccine and nonvaccine serotypes post–PCV13 introduction is required in Côte d’Ivoire to gain a full picture of the changing etiology of pediatric pneumococcal meningitis.

Of the *H. influenzae* isolates we serotyped, 2 were positively identified as Hib and both occurred during 2016, over 7 years after the introduction of the Hib vaccine in Côte d’Ivoire, with vaccine coverage rates estimated at 82% nationally (Supplementary Table 1) [10]. The remaining 4 isolates we serotyped were all non-Hib species (Figure 2). These results highlight that Hib is still responsible for some cases of PBM within children aged <5 years in Côte d’Ivoire despite the introduction of a conjugate vaccine. Only 2 of the children who were positive with *H. influenzae* infections had received a full course of the Hib vaccine, again suggesting that more work is needed within the country to ensure high levels of vaccine coverage and recording of vaccination status. Moreover, since the introduction of the Hib vaccine in South Africa, Kenya, and other East African regions, surveillance studies have reported meningitis cases caused by non-Hib serotypes, which could be due to a gradual reduction in the Hib population immunity as a result of the vaccine, leading to serotype replacement [35, 39–41]. Due to difficulties obtaining correct antisera and primers throughout the study, we were unable to serotype all of the *H. influenzae* samples received at the RRL (MRCG at LSHTM); thus, it is difficult to conclude that serotype replacement is occurring. However, the findings do show that among a total of 2762 suspected cases of meningitis, only 13 were caused by *H. influenzae* species, accounting for 0.47%, which is encouraging.

Interestingly, *N. meningitidis* was the least abundant pathogen seen in PBM, causing only 12 cases of PBM over the surveillance period. It is worth noting that 100% of these cases caused occurred in 2014, the same year that the MenAfriVac was introduced in Côte d’Ivoire, but, the MenAfriVac status of these patients was not recorded. However, since the vaccination campaign, there have been no cases of meningococcal meningitis reported in sentinel hospitals within Abidjan, providing support for the success of the vaccine at preventing meningococcal disease [42].

Microbiological culture of CSF samples is the current gold standard method used to detect the presence of pathogens causing meningitis. However, previous studies have shown that this has limited sensitivity, especially in patients who have received antibiotic treatment prior to CSF collection [20, 43, 44]. Data collected from the Global IB-VPD network have shown that the bacterial recovery rate from meningitis suspected cases by microbiology methods has been low [4, 45]. We found that at least 6.3% (175/2762) of suspected cases reported antibiotic use prior to admission, and for 52.6% (1453/2762) of the suspected cases, antibiotic use prior to admission was unknown. The 2 sentinel hospitals in Abidjan are large teaching hospitals, which receive referrals from primary and secondary healthcare facilities from the Abidjan district and surrounding Lagunes district whereby antibiotics may have been prescribed. Hence, antibiotic use prior to lumbar puncture may have been underreported and contributed to poor bacteriological recovery of pathogens in CSF samples.

There are limitations to this study. After diagnostic tests at the sentinel hospitals, only 41.9% (1160/2762) of CSF samples had sufficient quantity remaining to send to the RRL. Species-specific PCR assays could only be completed for 39.2% (1083/2762) of the total samples received due to insufficient volumes to allow for repeat analysis. In addition, a significant challenge encountered during this study was the absence of a patient identification number on some CSF samples, meaning that PCR data generated at MRCG at LSHTM could not be linked to clinical data at the sentinel site. This was a particular problem during the earlier years of the study and meant that some PCR data generated could not be added to the analysis presented here.

A further limitation of this study was that valuable information was not recorded for many patients. For instance, sequelae at discharge was not recorded for 92.6% (2557/2762) of patients with suspected meningitis and also not recorded for 81 of the 82 (98.7%) patients with confirmed PBM. The outcome at discharge was not recorded for 72.0% (1989/2762) of patients with suspected meningitis and 78% (64/82) of those with confirmed PBM. For future studies, it would be useful to obtain information regarding long-term sequelae and outcome at discharge, to determine accurate rates of morbidity and mortality associated with meningitis in the surveillance population, and to determine the level at which vaccination can help prevent this.

## CONCLUSIONS

Our surveillance revealed that pneumococcus was the main pathogen causing PBM before and after the introduction of PCV13 in Côte d’Ivoire. Conjugate vaccine serotypes were still detected post–vaccine introduction for both *S. pneumoniae* and *H. influenzae*. The high number of patients that reported antibiotic use before CSF collection may have contributed to the low culture recovery rate. We recommend enhanced surveillance within Côte d’Ivoire using highly sensitive methods such as PCR, to gain a full picture of the prevalence of IB-VPD and the serotypes circulating. Additionally, we recommend thorough reporting of vaccination status and potential morbidity associated with meningitis, to understand the effectiveness of the vaccines at reducing bacterial meningitis within children <5 years of age.

## Supplementary Data

Supplementary materials are available at *Clinical Infectious Diseases* online. Consisting of data provided by the authors to benefit the reader, the posted materials are not copyedited and are the sole responsibility of the authors, so questions or comments should be addressed to the corresponding author.

## Notes

***Invasive Bacterial Disease (IBD) Writing Group*** ***members.*** Brenda Kwambana-Adams, Madikay Senghore, Effua Usuf, Archibald Worwui, Egere Uzochukwu, Akram Zaman, Catherine Okoi, Florian Gehre, Leopold Tientcheu, Nuredin Ibrahim Mohammed, Felix Dube, Peter Ndow, Sambou Suso, Sheikh Jarju, Dam Khan, Chinelo Ebruke, Rowan Bancroft, Jason M. Mwenda, and Martin Antonio.

***Author contributions.*** M. A. and J. M. M. established the World Health Organization (WHO) Regional Office for Africa (AFRO)–supported Paediatric Bacterial Meningitis Surveillance Network in West Africa. M. A. supervised the overall network including setting up the sentinel surveillance system. C. C. B., H. K., and K. N. clinically investigated and recruited the patients at the sentinel sites, collected demographic data, performed microbiological testing, and shipped cerebrospinal fluid and bacterial isolates to Medical Research Council Unit The Gambia at the London School of Hygiene and Tropical Medicine (MRCG at LSHTM) for confirmatory testing and molecular analysis, supervised by B. K. A. and M. A. In addition, B. K. A. and M. A. developed the analysis plan and contributed to analysis and interpretation of data along with the IBD writing group. S. J., R. B., and B. K. A. drafted the manuscript along with C. B.-C. All authors contributed to the interpretation of the findings and the writing of the final manuscript.

***Acknowledgments.*** The authors thank the Ministry of Health of Côte d’Ivoire and the WHO Intercountry Support Team for coordination, advice, and support throughout the project; the surveillance team members as well as study participants and their families in Nigeria; the staff and students at the MRCG at LSHTM; and the IBD writing group for their advice and input.

***Financial support.*** Financial support for sentinel site surveillance was provided by the Cote d’Ivoire Ministry of Health, Gavi - the Vaccine Alliance, through a grant to the WHO for the African Paediatric Bacterial Meningitis Surveillance Network.

***Supplement sponsorship.*** This supplement was supported with funds from Gavi, the Vaccine Alliance through The World Health Organization and the CDC Foundation, and The Medical Research Council Unit The Gambia at the London School of Hygiene and Tropical Medicine.

***Potential conflicts of interest.*** E. U. served as a consultant for GlaxoSmithKline in 2014–2017, outside the submitted work. All other authors report no potential conflicts of interest. All authors have submitted the ICMJE Form for Disclosure of Potential Conflicts of Interest. Conflicts that the editors consider relevant to the content of the manuscript have been disclosed.
